# Pre-treatment consent framing shapes how laypeople evaluate CBT: a cross-cultural comparison of China and the United States

**DOI:** 10.3389/fpsyg.2026.1909096

**Published:** 2026-09-03

**Authors:** Yuanqing Wang, Feng Mei, Wei Zhao

**Affiliations:** 1 School of Educational Science, Jiangsu Second Normal University, Nanjing, Jiangsu, China; 2 Department of Medical Psychology, Brain Hospital Affiliated to Nanjing Medical University, Nanjing, Jiangsu, China; 3 Department of High-tech Business and Entrepreneurship, Faculty of BMS, University of Twente, Enschede, Netherlands

**Keywords:** CBT, common factors, cross-cultural, informed consent, IS-RSA, network analysis

## Abstract

People’s understanding of psychotherapy influences their treatment expectations and engagement. Informed consent is often one of the first formal processes through which this understanding is shaped. Yet, little cross-cultural research has examined its impact on lay people’s pre-treatment attitudes. This study investigated whether the perceived importance of common factors and cognitive behavioral therapy (CBT) techniques varies across cultural contexts and informed consent framing. Chinese (*n* = 297) and U.S. (*n* = 303) participants with no prior therapy experience were randomly assigned to standard CBT framing, enhanced framing that added information about common factors, or a non-framing (vignette-only) condition. They then used a 5-point Likert-type response format to rate the perceived importance of five elements for the success of CBT: empathy, working alliance, unconditional positive regard, expectancy, and CBT-specific techniques. Analyses combined ANOVA, Bayesian network analysis, and inter-subject representational similarity analysis (IS-RSA) to examine mean differences, attitude system structure, and individual profile similarity. Results indicated that culture influenced four of the five importance ratings. Chinese participants rated empathy and positive regard as more important, whereas U.S. participants rated working alliance and CBT techniques as more important (*ps* < 0.05). Attitude networks showed comparable global connectivity (*ΔM* = −0.254), although the empathy–positive regard association was stronger in the Chinese network. Enhanced framing produced higher importance ratings for empathy than standard framing, while standard framing produced higher ratings for CBT techniques (*ps* < 0.01). IS-RSA revealed that shared consent framing was more strongly associated with attitude profile similarity than shared cultural background (ρ_consent_ = 0.033, ρ_culture_ = 0.016), with the consent effect approximately twice the magnitude of the culture effect. Lay attitudes toward psychotherapy are affected by both cultural context and how treatment is initially presented. Informed consent may therefore serve an important communicative function that influences which therapy elements prospective clients view as important.

## Introduction

### Informed consent as pre-treatment psychoeducation

Psychotherapy does not begin with a technique; it begins with an explanation. Informed consent is an ethical and legal requirement for almost every form of psychological treatment (Trachsel et al., 2015). It also comes before treatment starts. For many clients, it is the first and often the only standard occasion on which a professional explains how therapy is meant to work. What that explanation emphasizes is therefore not a neutral choice, either ethically or clinically.

This role is best described as psychoeducational. Psychoeducation gives patients structured information about their condition and about why a treatment should help. It is widely used in mental health care and has been linked to better understanding of treatment, higher adherence, and fewer symptoms and relapses (Donker et al., 2009; Sarkhel et al., 2020). Work on role induction points in the same direction. Preparing clients for the therapy process clarifies what each side will do, and it lowers dropout (Oldham et al., 2012). Recent trials of improved consent procedures report similar gains in treatment expectations, motivation, and intentions to follow treatment (Gerke et al., 2024; Ladwig et al., 2024). These findings fit theories in which a credible and shared treatment rationale is itself part of what makes therapy work, because it helps make distress understandable and encourages hope and engagement (Kirsch, 1985; Wampold, 2015). Consent differs from other psychoeducation in one way. It is given before treatment, to people who have not yet decided whether therapy is for them.

Two problems make the content of this first message worth studying. First, it is not read in a cultural vacuum. The consent model itself rests on a particular view of the clinical encounter. In Chinese settings, treatment decisions have been described as more family-involved and more clinician-guided (Cong, 2004). Second, more information is not always better. An account that is one-sided or too optimistic may narrow how people understand therapy, and it may lead to disappointment once treatment begins (Rief and Glombiewski, 2017; Wells and Kaptchuk, 2012). It is therefore important to know what different consent framings actually tell lay audiences, and whether they tell the same thing in different cultural settings.

### CBT, common factors, and lay attitudes

Cognitive behavioral therapy (CBT) provides a useful context for these questions. It is among the most widely used and empirically supported psychotherapies for common mental health problems (Butler et al., 2006; Hofmann and Hayes, 2019), and it has been adapted successfully for Chinese and other East Asian populations (Hwang et al., 2015; Karat et al., 2022; Li et al., 2024). Its clinical importance and public familiarity make it a useful context for studying how laypeople understand psychotherapy before treatment begins.

Psychotherapy research distinguishes common factors from model-specific techniques. Frequently studied common factors include empathy, unconditional positive regard, working alliance, and treatment expectations, among others (Cuijpers et al., 2019; Wampold, 2015). In CBT, model-specific techniques include cognitive restructuring, behavioral activation, and exposure (Cohen et al., 2023; David et al., 2018). Separate meta-analyses have linked each of the four common factors to treatment outcome, with small to moderate effects (Constantino et al., 2018; Elliott et al., 2018; Farber et al., 2018; Flückiger et al., 2018). Public descriptions of CBT, however, usually focus on the techniques. Relational elements such as empathy and positive regard receive far less attention. Lay beliefs about how therapy works may follow the same emphasis.

Such beliefs matter for engagement. Positive expectations and perceived credibility predict better outcomes (Axelsson and Hedman-Lagerlöf, 2025; Pontén et al., 2024; Thielecke et al., 2024). Related research suggests that the fit between clients’ pre-treatment preferences and the treatment they receive may influence engagement and dropout (Lindhiem et al., 2014; Swift et al., 2021). Stigma and perceived mismatch with one’s assumptions can reduce acceptability (Eylem et al., 2020; Fischer et al., 2024; Gopalkrishnan, 2018). Yet most research has focused on mental health literacy and help-seeking (Eylem et al., 2020; Lam et al., 2015; Picco et al., 2016) rather than on how nonclinical laypeople evaluate specific therapy elements as contributors to treatment outcome.

### Social context and initial psychotherapy attitudes

Pre-treatment attitudes are shaped by broader social and cultural context (Fischer et al., 2024; Ryder et al., 2012; Wampold, 2015). Meta-analytic evidence shows that culturally adapted interventions outperform non-adapted ones, which suggests that the same content can mean different things to different audiences (Hall et al., 2016; Hwang et al., 2015). In the United States, CBT is widely presented as a structured, collaborative, skills-based treatment, which may make specific techniques and working alliance more noticeable to laypeople. In China, help-seeking is more strongly shaped by concerns about stigma, disclosure, and interpersonal trust (Gopalkrishnan, 2018; Lam et al., 2015). Relational safety and nonjudgmental acceptance may therefore matter more in early views of therapy. The structured, goal-focused nature of CBT may fit well with settings that value clear guidance and professional expertise (Huey et al., 2023; Li et al., 2024). Even so, relational elements may carry particular weight when people first consider whether therapy is acceptable at all.

Social context may also influence how attitudes are organized. If a therapy element carries particular meaning in a given setting, it may hold a more central place in the broader belief system (Borsboom, 2017; Dalege et al., 2016). The present study compares China and the United States as two social contexts in which psychotherapy may carry different public meanings. It does not directly measure cultural values, and observed differences should not be equated with fixed cultural traits.

### Consent framing and attitudes toward therapy elements

Within this broader psychoeducational role, the exact content of consent materials may direct attention toward different parts of therapy. Standard descriptions of CBT usually explain the treatment rationale and the specific techniques. They say less about empathy, acceptance, and other relational elements. Several authors have argued that consent belongs to good psychotherapeutic practice and can make treatment more understandable and credible to patients (Blease et al., 2018; Trachsel and Grosse Holtforth, 2019). In practice, however, therapists differ widely in how they handle consent, and it is often treated as a routine requirement rather than a clinically meaningful process (Eberle et al., 2021; Gerke et al., 2024).

Blease and Kelley (2018) provided direct experimental evidence that this emphasis matters. Standard CBT descriptions led laypeople to give more weight to the specific techniques. Descriptions that also explained the common factors led them to give more weight to relational elements. Three questions remain open. First, that study used a single national sample, so it is not known whether such framing effects hold in other cultural contexts, or whether culture and framing interact. Second, it compared group means. Means cannot show whether framing is related to the way evaluations of different therapy elements are linked to one another. Third, it did not test whether people who receive the same framing hold more similar overall attitude profiles than people who only share a cultural background.

### Interrelations and individual differences in psychotherapy attitudes

The last two of these questions cannot be answered by comparing group means alone. We therefore examined lay attitudes at three levels. Mean-level analyses show which therapy elements receive higher ratings. Psychometric Network Analysis (PNA) shows how the ratings are related to one another. Inter-Subject Representational Similarity Analysis (IS-RSA) compares participants’ overall response profiles.

The theoretical basis for viewing attitudes as interconnected systems comes from the Causal Attitude Network (CAN) model (Dalege et al., 2016). Rather than treating an attitude as a single latent trait, the CAN model views it as a network of connected evaluative reactions, such as beliefs, feelings, and behavioral tendencies toward an attitude object. Within such a network, evaluative reactions may differ in how closely they are connected to one another. Reactions with stronger or more numerous connections occupy more central positions in the attitude system. The connectivity of an attitude network has also been linked theoretically and empirically to attitude strength and stability (Dalege et al., 2016, 2019). A related network perspective has influenced psychopathology research, in which mental health problems are viewed as systems of connected symptoms rather than solely as manifestations of an underlying disorder (Borsboom, 2017). Although the present study does not directly test the causal assumptions of these models, this perspective provides a conceptual basis for examining therapy-related evaluations as an organized pattern rather than as isolated ratings.

This organization is relevant to treatment expectations. Expectations are not a single judgment but include beliefs about the rationale, the therapist, the process, and the likely outcome, which may be held with differing conviction (Laferton et al., 2017). Which of these is central should shape what a client notices and what the client treats as a sign of progress, and expectation violations are specific rather than global (Rief and Glombiewski, 2017). More strongly organized attitudes are also more stable and better predictors of behavior (Dalege et al., 2019). Two clients may therefore rate empathy and techniques as equally important, yet respond differently to a technique-focused first session.

Drawing on this perspective, PNA examines the conditional associations among evaluations of the five therapy elements and identifies which elements occupy more central positions in the network (Borsboom, 2017; Epskamp et al., 2012). Preference matching also concerns the fit between a client’s overall pattern of expectations and what a treatment offers, rather than an average across separate elements (Lindhiem et al., 2014; Swift et al., 2021). It therefore complements mean-level comparisons by showing how therapy-related evaluations are organized within each group. Because the present networks are based on cross-sectional data, centrality is interpreted descriptively rather than causally.

A separate but complementary question concerns similarity in participants’ overall attitude profiles. Mean-level and network analyses do not indicate whether participants who share a national context or pre-treatment framing condition show more similar patterns across all five ratings. IS-RSA addresses this question by preserving each participant’s full attitude profile and comparing profile similarity across participants (Kriegeskorte, 2008; Popal et al., 2019). In the present study, IS-RSA examines whether attitude-profile similarity is more strongly associated with shared national context or shared pre-treatment framing.

### The present study

This study examines how laypeople in China and the United States evaluate four common factors (therapist empathy, unconditional positive regard, working alliance, and expectancy) and one model-specific element (CBT-specific techniques) before treatment begins. It addresses four research questions:

#### Research question 1: group-level differences

Are cultural context and pre-treatment framing associated with importance ratings of specific therapy elements, and do these two factors interact?

*H1a* (Cultural-context main effects): We expected the China sample to rate empathy and unconditional positive regard as more important, and the U. S. sample to rate working alliance and CBT-specific techniques as more important.

*H1b* (Information type main effects): We expected enhanced framing to increase the perceived importance of relational elements and standard framing to increase the perceived importance of CBT-specific techniques.

Because no specific predictions were made about interactions between these two factors, the interaction analyses were exploratory.

#### Research question 2: attitude network pattern across cultures

Do attitude networks show different central elements across the two national samples?

*H2*: We expected empathy to be more central in the China sample and working alliance more central in the U.S. sample, with broadly similar overall structures.

#### Research question 3: pre-treatment framing and attitude network organization

Is pre-treatment framing associated with differences in the organization of attitudes?

*H3*: We expected framing differences to be linked to changes in network structure, particularly involving relational common factors and techniques. This part of the study remains partly exploratory due to limited subgroup power.

#### Research question 4: individual-level differences and similarity

Are individual attitudes more similar across national contexts or pre-treatment framing?

*H4*: We expected both factors to contribute but made no directional prediction about their relative strength.

## Materials and methods

### Participants

Participants were recruited online from the United States and China for a cross-cultural survey study on lay beliefs about the mechanisms of cognitive behavioral therapy (CBT). Participants in the United States were recruited through Amazon Mechanical Turk (MTurk; www.mturk.com) and completed the survey via an external link. These participants received a payment equivalent to an average hourly rate of approximately US$3.65. Participants in China were recruited and completed the survey directly on Wenjuanxing, an online survey platform widely used in China. They received 10 RMB after completing the survey. Both samples were convenience samples recruited through online platforms and are not representative of the general U.S. or Chinese populations. Demographic characteristics and statistical comparisons between the two samples are reported in Supplementary Table S3.

The initial sample comprised 337 U.S. participants and 331 Chinese participants. To be eligible, participants had to be at least 18 years old, belong to the national group from which they were recruited, and report no prior experience with psychotherapy. All participants provided written informed consent before taking part in the study.

The study protocol was approved by the Medical Ethics Committee of Nanjing Brain Hospital, with the approval number 2022-KY080-01. The study was conducted in accordance with the Declaration of Helsinki and relevant ethical regulations. During the study, participants’ rights and data security were strictly protected.

To ensure data quality, the screening process utilized an instructed-response attention check and a unified time-duration criterion. Since the U.S. and Chinese participants read equivalent materials in different languages, timing exclusions did not rely on specific English word or Chinese character counts. Instead, the timing limit applied to the total time participants spent reading the materials and answering the 20 questionnaire items. These comprised demographic and eligibility questions, five focal therapy-element importance-rating items, an instructed-response attention check, and other survey items; thus, only five items measured the primary study outcomes.

A minimum total-duration cutoff of 85 s was used to filter out excessively fast responses. This threshold combined the estimated reading time for the stimulus materials and the minimum response time for the rating items. The longest English text contained about 168 words. Based on an average reading speed of 238 words per minute (Brysbaert, 2019), this requires approximately 42 s. The 20 questionnaire items require a minimum of 2 s per item (Bowling et al., 2023), adding 40 s. The calculated minimum duration of 82 s was rounded to 85 s. Participants completing the task in less than 85 s were excluded.

The questionnaire also included one instructed-response attention check: “To show that you are reading carefully, please select ‘strongly disagree’ for this item”. Participants selecting any other option were excluded. In total, 68 respondents were excluded before the final analyses. This included 34 U.S. participants (10.1% of the initial sample) and 34 Chinese participants (10.3%). The final analytic sample included 303 U.S. participants and 297 Chinese participants.

In the final sample, 365 participants were female, 234 were male, and one participant did not report gender. Women accounted for 60.8% of the retained sample. The mean age was 30.00 years (SD = 10.70, range = 18–65). All participants had completed at least high school or an equivalent level of education, and 79.6% reported having a bachelor’s degree or higher. In addition, 61.3% reported being currently employed. The final sample was almost evenly distributed across the two national groups, with 50.5% from the United States and 49.5% from China.

### Procedure

All study procedures were administered online. After providing informed consent, participants first completed demographic questions and were then presented with a vignette describing a client experiencing psychological distress and considering CBT. Participants were then randomly assigned to one of three pre-treatment information conditions: standard framing, enhanced framing, or none framing. The standard framing condition described CBT primarily in terms of its treatment rationale and model-specific techniques, without explicitly describing common factors. The enhanced framing condition presented the same CBT-specific information and added descriptions of four common factors: therapist empathy, unconditional positive regard, working alliance, and treatment expectancy. Thus, “enhanced” refers only to the addition of common-factor information and does not imply that this condition was superior in quality. In the none framing condition, participants read the vignette without receiving any additional explanation of either CBT-specific techniques or common factors. The complete materials presented in the three conditions are provided in Appendix A of the Supplementary materials.

The study materials were adapted from the original English-language instrument developed by Blease and Kelley (2018) to assess lay beliefs about the components of successful psychotherapy. For use in the Chinese sample, the English materials were translated into Chinese by two bilingual translators: one doctoral student in English and one Chinese scholar trained in clinical psychology at the University of Manchester. The translated version was subsequently reviewed by one hospital-based mental health clinician and one university-based psychology PhD to assess the accuracy and appropriateness of the professional terminology. In addition, five lay respondents were invited to read the materials and comment on their comprehensibility; all indicated that the wording was easy to understand. This process was undertaken to improve the linguistic accuracy and face validity of the Chinese version.

Following the framing manipulation, participants rated five statements describing therapy elements that may contribute to successful CBT. Each statement assessed one element. Four statements assessed common factors—empathy, unconditional positive regard, working alliance, and treatment expectancy—and one assessed CBT-specific techniques. For example, the empathy statement read: “The therapist understands the client’s problems and feelings and can see things from the client’s perspective. The therapist communicates this understanding and shows concern for the client.”

Participants rated the perceived importance of each element to successful CBT using a 5-point Likert-type response format: 1 = not at all important, 2 = slightly important, 3 = moderately important, 4 = very important, and 5 = extremely important. Each element was assessed with a single descriptive item. These five item-level ratings, referred to throughout as importance ratings, were analyzed separately as the study outcomes. Where the broader term pre-treatment attitudes is used, it refers to the five importance ratings considered together. Consequently, internal consistency coefficients were not applicable. The complete instructions and wording of all five items are provided in Appendix B of the Supplementary materials.

### Data analysis

Data analysis proceeded in three stages. Mean-level differences were examined using two-way analyses of variance (ANOVAs), relations among the five therapy-element ratings were examined using Psychometric Network Analysis (PNA), and similarity among participants’ overall attitude profiles was examined using Inter-Subject Representational Similarity Analysis (IS-RSA).

#### ANOVA

Five separate between-subjects ANOVAs were conducted to examine ratings of the five therapy elements: empathy, unconditional positive regard, working alliance, treatment expectancy, and CBT-specific techniques. Each ANOVA used a 2 × 3 design. National context had two levels (China and the United States), and pre-treatment framing had three levels (standard framing, enhanced framing, and none framing).

The five ratings were analyzed separately because they represented conceptually distinct therapy elements and corresponded to element-specific hypotheses. Separate ANOVAs were therefore used instead of a MANOVA, as the analyses focused on the effects for each therapy element rather than on an overall multivariate effect. Each ANOVA tested the main effects of national context and pre-treatment framing, as well as their interaction.

Significant main effects of pre-treatment framing were followed by Bonferroni-adjusted pairwise comparisons. Significant interactions were examined using Bonferroni-adjusted simple-effects comparisons. Because no *a priori* hypotheses were specified for the interactions, these analyses were treated as exploratory. Effect sizes were reported as partial eta squared (ηp^2^), and statistical significance was evaluated using a two-sided alpha level of 0.05. The analyses were conducted using JASP 0.18.3.

#### Psychometric network analysis

The psychometric network analysis was conducted using JASP (Version 0.18.3). The network structure of psychological variables was estimated using a Bayesian Gaussian Graphical Model (GGM) (Williams and Mulder 2020). In this network, nodes represent the observed variables, and edges represent partial correlations. Each edge indicates the association between two nodes after controlling for all other measured nodes in the network. This procedure removes shared variance that is explained by those other nodes, including any construct overlap reflected in them. However, it cannot eliminate conceptual or wording overlap specific to a pair of items or account for unmeasured common factors. Edges should therefore be interpreted as conditional statistical associations rather than as evidence that the constructs are fully distinct or directly causally related.

The posterior distributions of the edge weights were estimated using a Markov chain Monte Carlo (MCMC) algorithm. To obtain a concise network structure and reduce the risk of false-positive edges, Bayes factors were used for edge selection. An edge was retained only when the Bayes factor provided strong evidence for an association over the null hypothesis of no association (BF₁₀ > 10; Kass and Raftery, 1995). Edges without sufficient evidence were constrained to zero, resulting in a sparse and interpretable network.

After estimating the network edges, we examined node centrality using strength, closeness, and betweenness centrality. These indices describe different aspects of the structural position of each therapy-element rating within the network (Epskamp et al., 2012). Strength centrality is the sum of the absolute partial-correlation weights of all edges connected to a node. In the present context, higher strength indicates that a therapy-element rating has stronger overall direct associations with the other ratings after controlling for the remaining measured nodes. High strength may reflect several moderately strong connections or a smaller number of particularly strong connections.

Betweenness centrality indicates how often a node lies on the shortest paths connecting other pairs of nodes. It therefore reflects the extent to which a therapy-element rating occupies a potential bridging position between different parts of the network. Closeness centrality reflects a node’s average shortest-path distance from all other nodes. Higher closeness indicates that the node occupies a more globally accessible position within the network.

In the present study, these indices were used to describe which therapy-element ratings occupied more central structural positions. They were not interpreted as evidence that any element was more clinically important or exerted a causal influence on the other ratings. Network robustness and stability were assessed through the accuracy of the posterior estimates. Bayesian analysis evaluates stability via the 95% Credible Intervals (CI) of the edge weights and centrality indices. A narrow credible interval that does not overlap with zero indicates that the estimated parameter is stable and reliable. All Bayesian analyses in JASP are based on the BGGM package (Williams and Mulder, 2020) in R, and network visualizations were generated using the qgraph package (Epskamp et al., 2012).

As recommended in recent methodological studies (Williams and Mulder, 2020), we investigated whether the network characteristics differed between groups. A Bayesian network comparison test was performed to assess differences in global strength (i.e., the absolute sum of all edge weights of the networks) and overall network structure (i.e., the posterior distributions of edge weights). Bayes Factors and 95% credible intervals were used to quantify the evidence for differences. These tests were performed using the R-package BGGM (Williams and Mulder, 2020).

#### Inter-subject representation similarity analysis (IS-RSA)

Inter-subject representational similarity analysis (IS-RSA) was conducted using custom scripts in Python. This method examines whether individuals who share a common characteristic (e.g., cultural background or informed consent condition) exhibit more similar patterns of attitudes toward common factors in CBT. Unlike variable-centered analyses, IS-RSA preserves each participant’s full response profile, allowing the analysis to capture similarity in overall attitude patterns rather than differences in individual variable means.

Representational dissimilarity matrices (RDMs) were constructed for the attitude profiles and for each grouping variable. First, a five-element attitude profile vector was formed for each participant from the importance ratings of the five therapy elements (Empathy, Working Alliance, Positive Regard, Expectancy, and CBT-Specific Techniques). Pairwise Spearman correlation was computed between each pair of participants’ profile vectors, and the Spearman distance matrix was defined as Dᵃᵗᵗ(i,j) = 1 − ρ_Spearman(aᵢ, aⱼ), yielding a 600 × 600 dissimilarity matrix. Second, a culture RDM was constructed using the absolute difference in cultural group membership: Dᶜᵘˡᵗ(i,j) = |cultureᵢ − cultureⱼ|, taking values of 0 (same culture) or 1 (different culture). Third, an informed consent condition RDM was similarly defined as Dᶜ°ⁿᵈ(i,j) = |conditionᵢ − conditionⱼ|, taking values of 0 (same condition), 1 (adjacent conditions), or 2 (extreme conditions: Standard vs. None).

Weighted composite predictor matrices were formed as linear combinations of the culture and condition RDMs: P(α) = α·Dᶜᵘˡᵗ + (1 − α)·Dᶜ°ⁿᵈ, where α was varied from 0 (pure consent framing) to 1 (pure culture) in increments of 0.05 (21 models). For each weight level, the Spearman rank correlation was computed between the lower-triangular entries of the composite predictor matrix P(α) and the corresponding entries of the attitude RDM Dᵃᵗᵗ. Statistical significance was assessed via a permutation test with 10,000 iterations. In each iteration, participant indices were randomly shuffled, the attitude RDM was recomputed from the permuted order, and the Spearman correlation was recalculated. The permutation *p*-value was computed as the proportion of permuted correlations that equaled or exceeded the observed correlation. All Spearman correlations were Fisher z-transformed for effect size reporting.

To control for multiple comparisons across the 21 weight levels, Bonferroni correction was applied: corrected *p*-values were calculated by multiplying the raw permutation *p*-values by 21 and capping at 1. This yielded a weighted curve of IS-RSA prediction performance across different combinations of culture and consent framing weights, allowing us to estimate the relative contribution of each factor to individual-level variability in CBT attitudes.

## Results

### Significant differences in attitudes toward common factors in CBT across eastern and Western cultures

Five separate two-way ANOVAs were used to examine the perceived importance of empathy, expectancy, unconditional positive regard, CBT-specific techniques, and working alliance. Each analysis tested the main and interaction effects of national context (China vs. the United States) and pre-treatment framing (enhanced, standard, or none framing). Significant effects were followed by Bonferroni-adjusted *post hoc* comparisons. Interaction analyses were exploratory because no interaction effects were hypothesized *a priori* (see Table 1; Supplementary Table S1).

**Table 1:** Descriptive statistics and ANOVA results for attitudes toward CBT therapy elements.

DV	East Standard	East Enhanced	East None	West Standard	West Enhanced	West None	F culture	η^2^ culture	F info	η^2^ info	F Inter	η^2^ Inter
Empathy	3.39 (1.38)	3.75 (1.23)	3.83 (1.24)	3.18 (1.29)	3.73 (1.28)	3.33 (1.30)	5.37	0.0087	6.36	0.0210	1.78	0.0058
Working alliance	2.83 (1.35)	3.36 (1.32)	3.21 (1.38)	3.56 (1.02)	3.40 (1.32)	3.29 (1.46)	6.97	0.0116	1.06	0.0036	4.34	0.0144
Positive regard	2.62 (1.17)	3.04 (1.37)	2.90 (1.22)	2.22 (1.08)	2.92 (1.31)	2.59 (1.29)	7.62	0.0127	10.18	0.0331	0.67	0.0022
Expectancy	2.36 (1.26)	2.43 (1.29)	2.19 (1.28)	2.00 (1.22)	2.30 (1.31)	2.37 (1.31)	0.98	0.0016	1.10	0.0037	2.24	0.0074
CBT techniques	3.80 (1.43)	2.41 (1.39)	2.85 (1.44)	4.04 (1.29)	2.65 (1.41)	3.41 (1.40)	9.11	0.0141	49.69	0.1433	0.91	0.0031

DV, dependent variable; Info, information type; Inter, interaction. The number format in the condition column is mean (standard deviation).

**Figure 1:**
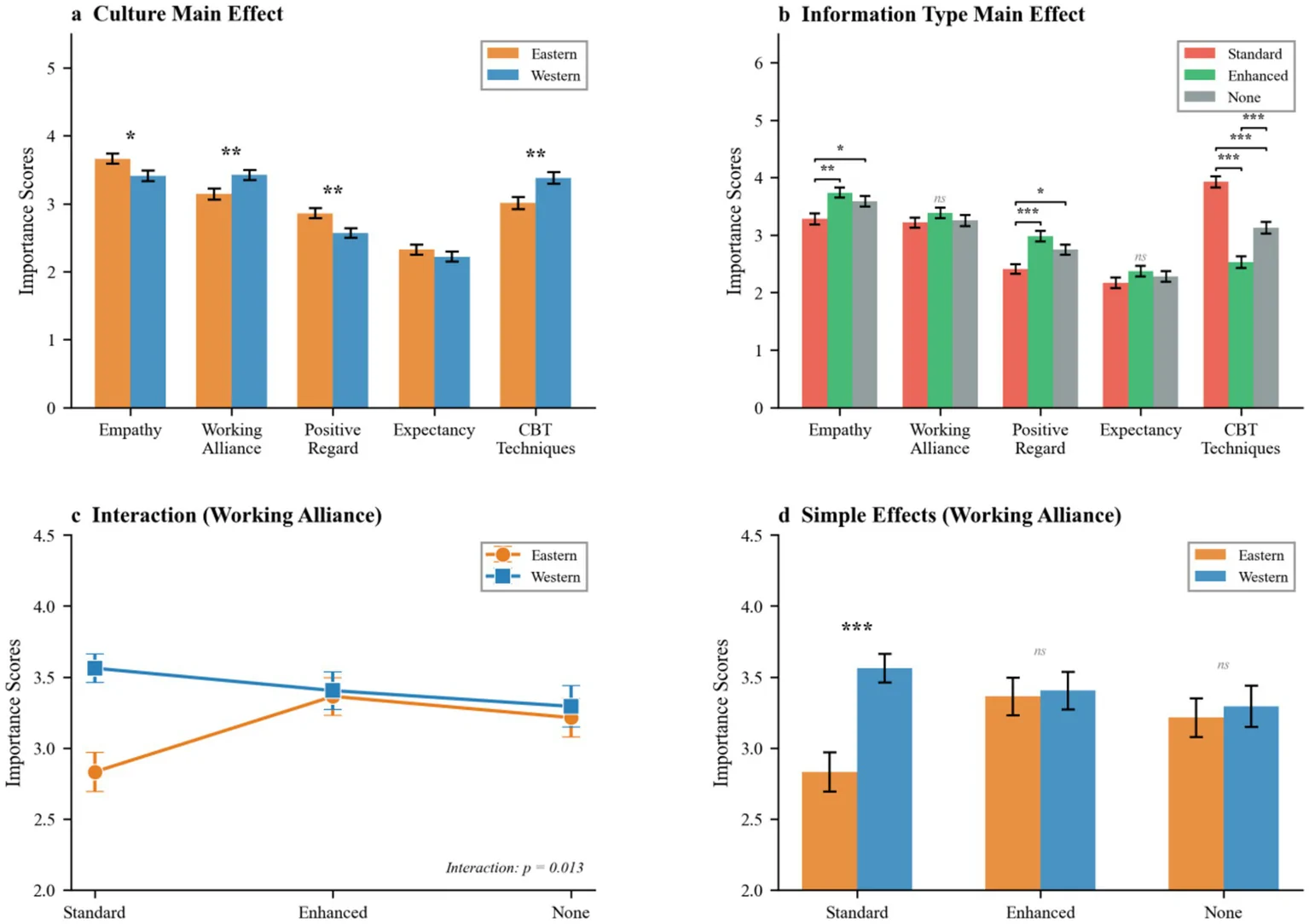
ANOVA results for culture and information type effects on attitudes toward CBT therapy elements. **(a)** Culture main effect: Mean importance ratings for five therapy elements across Eastern and Western participants. Error bars represent ±1 SE. **(b)** Information type main effect: Mean importance ratings across three pre-treatment framing conditions (Standard, Enhanced, None). * *p* < 0.05,** *p* < 0.01,***p < 0.001. **(c)** Culture × Information type interaction for Working Alliance. **(d)** Simple effects of culture within each information type condition for Working Alliance.

For Empathy, there was a significant main effect of cultural context (*F_(1, 594)_* = 5.37, *p* = 0.02, *η^2^* = 0.009), with Eastern participants rating empathy as significantly more important than Western participants (*t_(594)_* = 2.32, *p* = 0.02, Figure 1a). A significant main effect of information type was also observed (*F_(2, 594)_* = 6.36, *p* = 0.002, *η^2^* = 0.021), and participants in the standard consent group gave significantly lower importance ratings than those in the enhanced consent group (*t_(594)_* = −3.50, *p* = 0.002, Figure 1b). The interaction effect was not significant (*F_(2, 594)_* = 1.78, *p* = 0.17, *η^2^* = 0.006, Supplementary Figures S1a,b).

For Expectancy, no significant effects were observed: cultural context (*F_(1, 594)_* = 0.98, *p* = 0.32, *η^2^* = 0.002, Figure 1a), information type (*F_(2, 594)_* = 1.10, *p* = 0.33, *η^2^* = 0.004, Figure 1b), or their interaction (*F_(2, 594)_* = 2.24, *p* = 0.11, *η^2^* = 0.007, Supplementary Figures S1e,f).

For Unconditional Positive Regard, the main effect of cultural context was significant (*F_(1, 594)_* = 7.62, *p* = 0.006, *η^2^* = 0.013), with Eastern participants rating positive regard as significantly more important than Western participants (*t_(594)_* = 2.76, *p* = 0.006, Figure 1a). The main effect of information type was also significant (*F_(2, 594)_* = 10.18, *p* < 0.001, *η^2^* = 0.033), and the standard consent group was significantly lower than both the enhanced (*t_(594)_* = −4.49, *p* < 0.001) and no-consent (*t_(594)_* = −2.62, *p* = 0.03) groups (Figure 1b). The interaction was not significant (*F_(2, 594)_* = 0.67, *p* = 0.51, *η^2^* = 0.002, Supplementary Figures S1c,d).

For Specific CBT Techniques, a significant main effect of cultural context was observed (*F_(1, 594)_* = 9.11, *p* = 0.003, *η^2^* = 0.015), with Western participants rating CBT techniques as more important than Eastern participants (*t_(594)_* = 3.02, *p* = 0.003, Figure 1a). A strong main effect of information type was found (*F_(2, 594)_* = 49.69, *p* < 0.001, *η^2^* = 0.143, Figure 1b), with the standard consent group scoring significantly higher than both the enhanced (*t_(594)_* = 9.94, *p* < 0.001) and no-consent (*t_(594)_* = 5.64, *p* < 0.001) group. Additionally, the no-consent group was higher than the enhanced group (t_(594)_ = 4.34, *p* < 0.001). No interaction was found (*F_(2, 594)_* = 0.91, *p* = 0.40, *η^2^* = 0.003, Supplementary Figures S1g,h).

For Working Alliance, the main effect of cultural context was significant (*F_(1, 594)_* = 6.97, *p* = 0.009, *η^2^* = 0.012), with Western participants rating working alliance as more important than Eastern participants (*t_(594)_* = 2.64, *p* = 0.009, Figure 1a). The main effect of information type was not significant (*F_(2, 594)_* = 1.06, *p* = 0.35, *η^2^* = 0.004, Figure 1b), but the interaction was significant (*F_(2, 594)_* = 4.34, *p* = 0.01, *η^2^* = 0.014, Figures 1c,d). Follow-up comparisons showed that U.S. participants rated working alliance as significantly more important than Chinese participants under standard framing, t₍₅₉₄₎ = −3.93, *p* < 0.001. This cultural difference was not significant under enhanced framing, t₍₅₉₄₎ = −0.22, *p* = 0.83, or under none framing, t₍₅₉₄₎ = −0.43, *p* = 0.67 (Figure 1d).

In summary, cultural context significantly influenced importance ratings of Empathy, Unconditional Positive Regard, Specific Techniques, and Working Alliance, but not Expectancy. Eastern participants rated Empathy and Unconditional Positive Regard as more important, whereas Western participants rated Specific Techniques and Working Alliance as more important. Information type significantly influenced importance ratings of Empathy, Unconditional Positive Regard, and Specific Techniques. No main effect of information type was observed for Expectancy or Working Alliance, although Working Alliance showed a significant interaction between cultural context and information type.

### Cross-cultural and consent-based attitude networks of common factors in CBT

**Figure 2:**
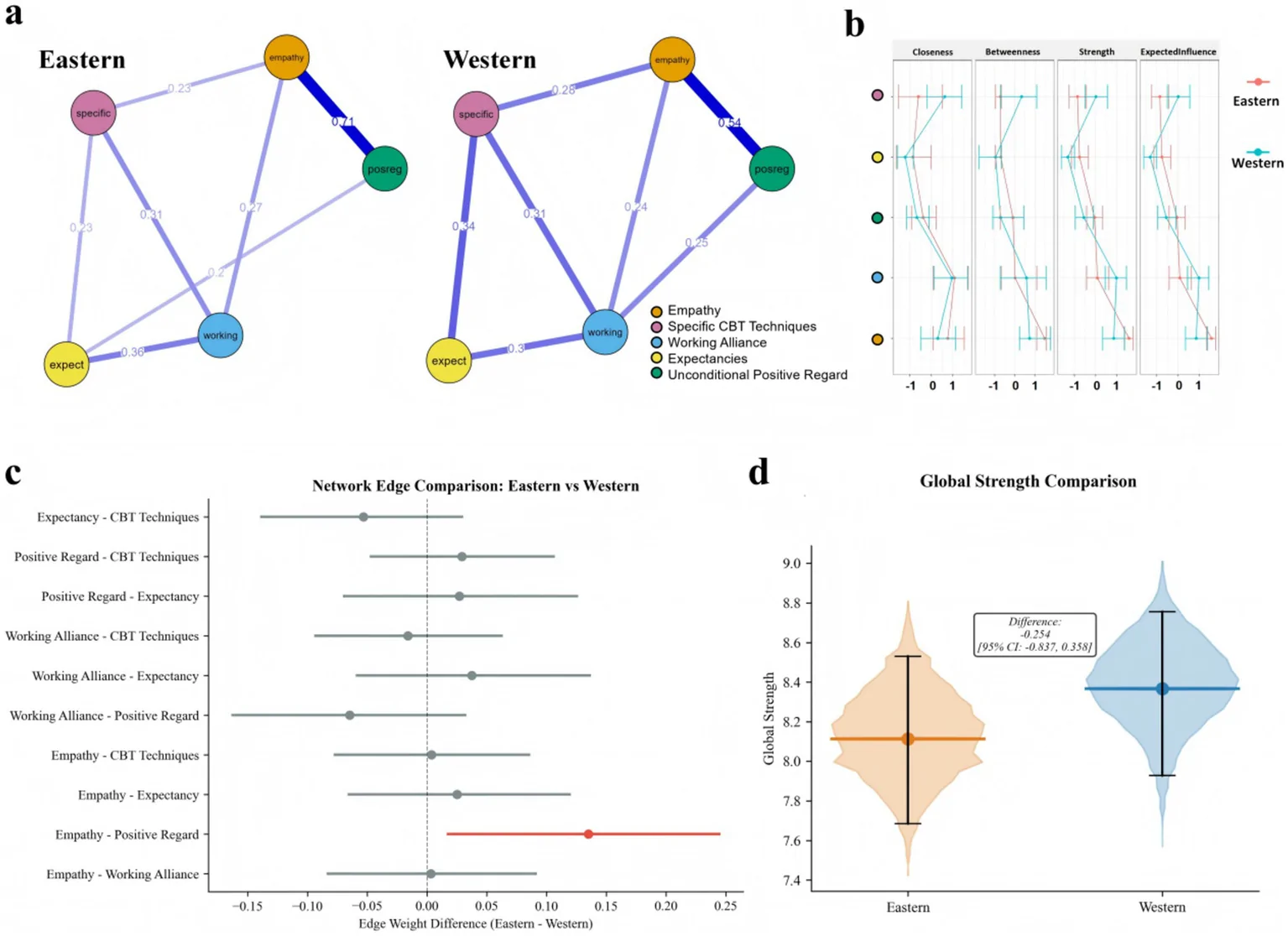
Bayesian network analysis of attitudes toward CBT therapy elements. **(a)** Eastern attitude network (*n* = 297) and Western attitude network (*n* = 303). Edge widths reflect partial correlation magnitudes; only edges with Bayes factors indicating substantial evidence are shown. **(b)** Centrality comparison: strength centrality *z*-scores across the five nodes for Eastern and Western networks. **(c)** Network edge comparison between Eastern and Western attitude network. **(d)** Global strength comparison between Eastern and Western attitude network.

To investigate the structural relationships among attitudes toward CBT common factors, we conducted a series of Bayesian Gaussian graphical models (GGMs) within a network analysis framework. Participants were first divided into Eastern and Western cultural groups, and within each group, further stratified by the type of informed consent received (standard, enhanced, or none). A Bayesian network comparison test between the Eastern and Western networks revealed no significant differences in global strength (*ΔM* = −0.254, *95% CI* [−0.837, 0.358], Figure 2d), indicating that the overall level of connectivity in the attitude networks was similar across the two cultural groups.

#### Network structure

Edge-level comparison between the two groups indicated that 9 of 10 edges did not differ significantly (*95% CI*s all crossed zero), with the exception of the Empathy–Positive regard edge, which showed a stronger association in the Eastern network (*Δ_eastern-western_* = 0.135*, 95% CI* [0.018, 0.244], Figure 2c). In the Western cultural group (see Figure 2a right), the attitude network was fully connected and showed moderate overall density. The strongest edge was between Empathy and Positive regard (partial correlation = 0.54), followed by Expectancy and Specific Techniques (partial correlation = 0.34), and Specific Techniques and Working Alliance (partial correlation = 0.30). No edge was observed between Expectancy and Positive Regard.

Subgroup analyses by informed consent type in the Western group revealed different network structures across three conditions (see Supplementary Figure S2b). The Empathy-Working Alliance edge was present in the standard consent subgroup, but absent in the no-consent subgroup, whereas the Empathy-Specific Techniques edge showed the opposite pattern. Notably, in the enhanced consent subgroup, both Empathy-Working Alliance and Empathy-Specific Techniques edges were present. The Working Alliance-Specific Techniques edge appeared in the no-consent subgroup, but was absent in the enhanced consent subgroup. In contrast, the Working Alliance-Expectancy edge was present in the enhanced consent subgroup, but not in the no-consent subgroup. In the standard consent subgroup, both the Working Alliance-Specific Techniques and Working Alliance-Expectancy edges were observed.

In the Eastern group, the overall network was also fully connected, but showed subtle differences in configuration (see Figure 2a left). The strongest edge also appeared between Empathy and Positive regard (partial correlation = 0.71), followed by Expectancy and Working Alliance (0.36), and Specific Techniques and Working Alliance (0.31). Unlike the Western network, no edge was observed between Positive Regard and Working Alliance, whereas an edge was present between Positive Regard and Expectancy.

Descriptive subgroup analyses within the Eastern sample suggested further differences across informed-consent conditions (see Supplementary Figure S2a). The standard consent subgroup showed the densest network (sparsity = 0.40), with Empathy–Positive Regard (0.66) and Specific Techniques–Working Alliance (partial correlation = 0.51) remaining the dominant edges. In the standard consent subgroup, no edge was found between Empathy and Working Alliance, whereas this edge was present in the enhanced consent subgroup. Conversely, an edge between Empathy and Specific Techniques was present in the standard consent subgroup, but not in the enhanced consent subgroup. In the no-consent subgroup, Empathy showed edges with both Working Alliance and Specific Techniques. An edge between Expectancy and Positive Regard was observed in the standard consent subgroup but was absent in the no-consent subgroup. By contrast, Expectancy was connected to Specific Techniques only in the no-consent subgroup. In the enhanced consent subgroup, Expectancy showed no edge with either Positive Regard or Specific Techniques.

#### Centrality indices

In the Eastern network (see Figure 2b), empathy emerged as the most central node across all three indices, with the highest strength centrality (*z =* 1.57), closeness (*z =* 0.77), and betweenness (*z =* 1.49). Working Alliance ranked second in strength (*z =* 0.89), while Specific Techniques (*z =* −0.48) and Positive Regard (*z =* −0.17) occupied intermediate positions. Expectancy was consistently the most peripheral node (strength *z =* −0.75), suggesting that treatment outcome expectations, though rated as moderately important at the mean level, are not tightly coupled with other therapy-related attitudes in this group. Centrality indices across the three informed consent conditions were broadly consistent (see Supplementary Figure S3a), with empathy holding the highest strength centrality in all three subgroups (Standard: *z =* 1.07; Enhanced: *z =* 1.09; No-consent: *z =* 1.55). Working Alliance showed a marked sensitivity to condition: its betweenness centrality rose sharply from the Standard (*z =* −0.04) to the Enhanced subgroup (*z =* 1.14), before moderating in no-consent subgroup (*z =* 0.35). A similar dynamic was observed for Specific Techniques, whose betweenness declined from Standard (*z =* 0.25) to Enhanced subgroup (*z =* −0.69) and partially recovered in No-consent subgroup(*z =* −0.39). Expectancy remained the most peripheral node in all three conditions. The Enhanced subgroup produced the most evenly distributed centrality profile across nodes, whereas No-consent subgroup yielded the most concentrated structure, favouring empathy.

In the Western network (see Figure 2b), Working Alliance was the most central node in strength (*z =* 0.98), followed by empathy (*z =* 0.86). Specific Techniques held a near-average position (*z =* 0.02), in contrast to its more peripheral role in the Eastern network. Expectancy was again the least central node (strength *z =* −1.08), mirroring the Eastern pattern. The centrality pattern varied across conditions (see Supplementary Figure S3b). Under the Standard subgroup, Working Alliance held the highest strength centrality (*z =* 0.94) and the highest betweenness (*z =* 1.09), with empathy in a secondary role (strength *z =* 0.31). Under the Enhanced subgroup, this hierarchy reversed that empathy became dominant (strength *z =* 1.37, betweenness *z =* 0.85), while Working Alliance declined (strength *z =* 0.55) and Specific Techniques dropped sharply (strength *z =* −0.77). Under the No-consent subgroup, Specific Techniques unexpectedly became the most central node (strength *z =* 1.05, betweenness *z =* 0.98), with empathy (*z =* 0.53) and Working Alliance (*z =* −0.49) in reduced positions. This pronounced cross-condition variability in the Western sample contrasts with the relative stability observed in the Eastern sample and suggests that the Western attitude network may be more sensitive to the content of pre-treatment information.

### Cultural and consent-based individual differences patterns in CBT attitudes

**Figure 3:**
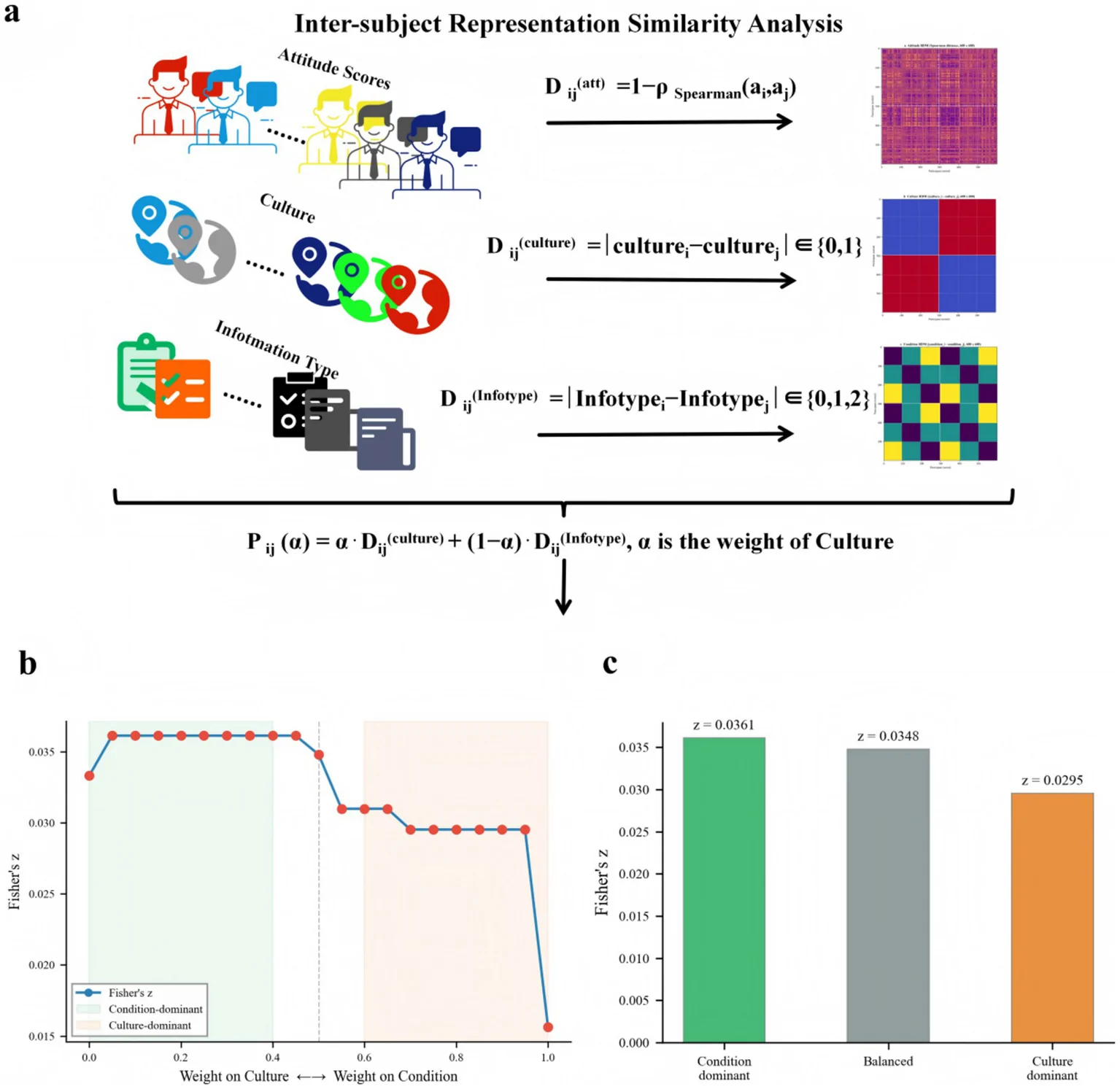
Inter-subject representational similarity analysis (IS-RSA). **(a)** Attitude representational dissimilarity matrix (RDM) based on Spearman distance between individual attitude profiles; Culture RDM based on absolute difference in cultural group membership (0 = same culture, 1 = different); Condition RDM based on absolute difference in informed consent condition (0 = same, 1 = adjacent, 2 = extreme). **(b)** Weighted combination curve showing Fisher *z*-transformed Spearman correlations across culture-condition weight combinations. Shaded areas indicate condition-dominant (green) and culture-dominant (orange) regions. **(c)** Model comparison bar chart showing Fisher *z* values for the condition-dominant, balanced, and culture-dominant models.

To examine how cultural background and informed consent type jointly influence individual differences in attitudes toward common factors in CBT, we employed inter-subject representational similarity analysis (IS-RSA). This approach tests whether individuals sharing a similar cultural background or informed consent condition exhibit more similar attitudinal patterns at the level of individual response profiles. By systematically varying the relative weighting of culture and consent within the composite predictor RDM, we compared their contributions to the representational structure of individual attitudes (see Figure 3a).

The condition-only model (α = 0) yielded a significant but modest effect (*ρ* = 0.033, *Fisher-z =* 0.033, *p* < 0.001), as did the culture-only model (α = 1; ρ = 0.016, *Fisher-z =* 0.016, *p* < 0.001). Models with dominant weighting of informed consent (α ≤ 0.4) produced the strongest and most stable similarity effects (*Fisher-z =* 0.036, all *p* < 0.001). When culture and consent were balanced (α = 0.5), the effect decreased slightly (*ρ* = 0.035, *Fisher-z =* 0.035, *p* < 0.001). As the weighting shifted toward culture (α ≥ 0.6), the effect continued to decrease monotonically, reaching its minimum at the culture-only model (*ρ* = 0.016) (see Figure 3b). All 21 weight combinations were statistically significant after Bonferroni correction (all *p_corrected* < 0.01, Supplementary Table S2).

The IS-RSA findings indicate a monotonic decrease in profile similarity as the weighting shifted from consent-dominant to culture-dominant models, with no evidence of a non-linear or inverted-U pattern. The effect of consent framing was approximately twice as large as the effect of culture (ratio of *Fisher-z* values = 2.1:1, Figure 3c).

In sum, these findings suggest that while both culture and informed consent contribute to individual differences in attitudes toward CBT, the type of informed consent framing exerts a stronger influence on attitude profile similarity than cultural background alone. This indicates that the contextual framework provided by pre-treatment information may shape individuals’ patterns of therapy-related attitudes in ways that are detectable across cultural contexts. Although cultural variation exists in mean-level attitudes toward common factors in CBT, the organizing influence of informed consent type on attitude profiles appears to be relatively consistent across both Eastern and Western samples.

## Discussion

This study examined how laypeople with no prior psychotherapy experience in China and the United States evaluated key therapy elements related to CBT in a hypothetical treatment scenario. Overall, the findings were broadly consistent with the main hypotheses, although support differed across questions and methods.

### Cross-culture differences in attitudes toward therapy elements

The clearest cross-national differences concerned four therapy elements: the China sample rated empathy and unconditional positive regard as more important, whereas the U.S. sample rated working alliance and CBT-specific techniques as more important. Network analysis complemented these findings: empathy was most central in the China sample and working alliance in the U.S. sample. Social context was thus associated not only with what people value but also with which elements they use as a reference point when thinking about therapy.

These differences may reflect socially shaped assumptions about what makes psychotherapy acceptable, trustworthy, and effective (Fischer et al., 2024; Ryder et al., 2012; Wampold, 2015). Where stigma, self-disclosure concerns, and interpersonal trust are salient, relational safety may matter more (Gopalkrishnan, 2018; Lam et al., 2015), which may explain the higher empathy and positive-regard ratings in the China sample. In U.S. discourse, by contrast, CBT is often presented as structured, goal-oriented, skills-based, and collaborative (Cohen et al., 2023; David et al., 2018; Hofmann and Hayes, 2019), which may make working alliance and techniques more visible to laypeople. This interpretation is consistent with Blease and Kelley (2018), whose U.S.-based study showed that making common factors explicit increased the weight given to relational elements; our finding that framing effects did not differ between samples suggests the pattern is not limited to a Western sample. The findings also fit evidence that CBT is effective in Chinese and East Asian settings (Karat et al., 2022; Li et al., 2024; Ng and Wong, 2018), and that its structured, goal-focused features may suit settings that value guidance and professional expertise (Huey et al., 2023). Replication in more diverse East Asian and Western samples is needed before broader cross-cultural conclusions can be drawn.

These interpretations should be treated with caution. We did not directly assess therapy literacy, self-disclosure concerns, trust in professionals, or cultural values such as collectivism and power distance; differences may also reflect familiarity with psychotherapy terminology, translation, sample composition, or response styles. The findings therefore indicate cross-national differences between the present samples rather than fixed cultural traits. Working alliance in particular referred to lay views of therapist–client cooperation in a hypothetical scenario, not an alliance formed during therapy; its higher rating in the U.S. sample likely reflects the greater visibility of collaboration in lay understandings of CBT.

### Cross-culture similarities and the meaning of the expectancy finding

The findings also showed meaningful similarities. Expectancy did not differ significantly across national context or framing; this null finding should be interpreted cautiously, as it may reflect both substantive and methodological factors.

Substantively, expectancy concerns a general belief in the possibility of treatment benefit rather than a specific therapist behavior or procedure, and it has been linked to outcomes across treatment formats (Axelsson and Hedman-Lagerlöf, 2025; Constantino et al., 2018). Because it reflects a broad orientation toward possible improvement, it may be less sensitive than the other elements to brief descriptions of how CBT works. Methodologically, the scenarios gave expectancy little attention, so participants may have attended to it less and the manipulation may have had less effect on their ratings. Expectancy is not the placebo effect but is often considered one of its processes (Enck and Zipfel, 2019); lay participants may therefore have viewed it as a general or nonspecific influence rather than a distinct element of CBT. Future studies should distinguish outcome expectancy, treatment credibility, general hope for improvement, and confidence in a specific treatment rationale. The network findings also revealed a shared pattern: in both networks, empathy and unconditional positive regard remained strongly associated after adjustment for the other ratings, consistent with research viewing them as closely related aspects of the therapeutic relationship (Elliott et al., 2018). Lay evaluations may thus contain both shared and context-sensitive components: people may recognize similar therapy elements but differ in the importance and centrality assigned to them.

### The role of the informed consent framework in considering individual differences

The framing effects point to a function of informed consent that is less often discussed: how CBT was described was more closely related to attitude profiles than cultural context was. The enhanced condition increased the perceived importance of empathy relative to standard framing, whereas standard framing produced the highest ratings of CBT-specific techniques (Blease and Kelley, 2018). Unconditional positive regard was rated lower under standard framing than under both enhanced and none framing; framing had no main effects on expectancy or working alliance, although an exploratory culture-by-framing interaction emerged for working alliance.

These effects extend earlier work linking consent procedures to motivation and adherence (Ladwig et al., 2024). Gerke et al. (2024) found that an optimized consent consultation increased autonomous motivation and adherence intentions, although its additional elements preclude attributing the effect to framing alone, and the present study did not assess motivation. More broadly, informed consent is part of good psychotherapeutic practice rather than an administrative task (Eberle et al., 2021); when ethically conducted, it may make treatment more understandable, credible, and personally relevant, strengthening patients’ meaning responses (Trachsel and Grosse Holtforth, 2019). The present findings suggest that even brief pre-treatment descriptions may shape which therapy elements laypeople consider important before any direct treatment experience.

Beyond mean-level differences, the exploratory network results suggest that framing may also relate to how these ratings are organized. In the Chinese sample, empathy remained the most central node across all three conditions, a stable relational focus. In the U.S. sample, the central element shifted from working alliance under standard framing to empathy under enhanced framing and CBT techniques in the none framing control. These shifts align with H3 and suggest that how treatment is first presented may relate to how prospective clients link relational and technical beliefs.

These findings do not explain why framing was associated with the differences. We did not assess comprehension, recall, perceived credibility, trust, or emotional responses to the materials. We therefore cannot determine whether the differences reflected understanding, salience, plausibility, or other processes. The positive-regard finding also requires caution, as it did not show a simple enhanced-versus-standard contrast, possibly because positive regard is more abstract than empathy and overlaps with general perceptions of therapist warmth. Future studies should use comprehension checks and examine whether some common factors are easier to communicate in public-facing materials.

IS-RSA findings pointed in the same direction at the individual level, although the effects were modest: shared framing was associated with more similar overall profiles than shared national context, with the framing effect approximately twice the size of the culture effect (all 21 weighted combinations significant after Bonferroni correction). Given the small effect sizes, these results are best viewed as preliminary evidence that shared framing is modestly associated with similarity in overall therapy-related evaluations.

How CBT is described at first contact may therefore shape prospective clients’ treatment expectations, and consent materials can serve as psychoeducation. Because this study did not assess treatment expectations or behavior, these possible effects require further research.

### Methodological and practical implications

The study also has methodological value. Most research on public attitudes toward psychotherapy relies on mean-level comparisons, which cannot capture all differences across groups or conditions. Combining them with psychometric network analysis and IS-RSA yielded three complementary levels—average importance, group-level structure, and individual profile similarity—that provided complementary rather than interchangeable information: an element could occupy a central network position without receiving the highest mean rating, and shared framing could relate to profile similarity even when its effects on individual ratings were modest.

These methods require cautious interpretation. Network centrality is descriptive and does not establish causal mechanisms, and the similarity associations were small and partly dependent on analytic choices. The practical implications are also limited—the findings do not show that changing consent materials improves uptake, alliance, retention, or clinical outcomes, only that early treatment descriptions may shape what prospective clients notice and value.

This may matter in service websites, intake materials, psychoeducation documents, and consent forms. If CBT is presented only in technical terms, potential clients may view treatment mainly as a set of procedures. A description that also covers empathy, acceptance, and collaborative work may provide a more complete account, since relational processes also contribute to outcome (Cuijpers et al., 2019). A technique-only account therefore provides incomplete information for informed consent (Blease et al., 2018). Balanced descriptions may also reduce mismatches between expectations and early sessions, so materials should match the treatment actually delivered. Clear information about treatment has been associated with better understanding and adherence (Donker et al., 2009; Oldham et al., 2012), although not examined here. These considerations may matter most in culturally diverse settings, where different weights on relational and technical elements suggest that first-contact materials should not be treated as neutral or one-size-fits-all; at a minimum, they should communicate both relational and technical aspects clearly while accurately reflecting the treatment to be delivered.

## Limitations and future directions

Several limitations should be noted. Both samples were online convenience samples, and participants evaluated therapy in a hypothetical scenario rather than as prospective clients. Screening out participants with prior psychotherapy experience helped isolate initial evaluations but limits generalizability to treatment-seeking and therapy-experienced populations, whose established attitudes may be less responsive to framing and may depend on the valence of prior experiences. There was also no pre-test, so we could not assess within-person change; although randomization supports baseline comparability, it does not verify it, and future studies should assess prior attitudes in a separate session or with parallel measures. Future research should also test whether importance ratings predict treatment uptake, early alliance, retention, or symptom change.

We also did not record participants’ own current or past psychological problems, which could affect identification with the client in the vignette and ratings of empathy and acceptance; future studies should include a brief screening measure as a covariate. The vignette described depression only, and ratings may differ for other presenting problems—techniques may seem more important for anxiety disorders, and empathy and acceptance for grief. Whether presenting problem interacts with cultural context or framing is unknown; varying the problem across vignettes would allow this to be tested.

Finally, several explanatory variables were not measured. These include therapy literacy, comprehension of the materials, perceived credibility, and trust in mental health professionals. We also compared two national settings without measuring cultural values or testing measurement invariance across language versions. The findings should therefore not be read as direct evidence of cultural mechanisms. Future work could add direct measures of values, stigma, trust, and beliefs about authority, and test whether the measures work similarly across groups (Putnick and Bornstein, 2016).

## Conclusion

This study found that lay evaluations of CBT-related therapy elements varied by both national context and pre-treatment informed consent framing. Participants in the China sample gave higher importance ratings to therapist empathy and unconditional positive regard, whereas participants in the U.S. sample gave higher ratings to working alliance and CBT-specific techniques. The network analyses indicated that these differences were not limited to mean ratings. Although the two national networks showed similar overall connectivity, empathy was the most central element in the China network, while working alliance was most central in the U.S. network.

Pre-treatment framing was also linked to differences in lay attitudes. Enhanced framing increased the perceived importance of empathy, whereas standard CBT framing produced the highest ratings of CBT-specific techniques. The IS-RSA results further showed that shared framing predicted similarity in individual attitude profiles more strongly than shared national context. The framing effect was approximately twice as large as the culture effect, although both effects were small. Exploratory network analyses also suggested that framing may be related to the organization of therapy-related attitudes.

Taken together, these findings indicate that informed consent may function not only as an ethical requirement, but also as an early form of clinical communication. The way CBT is first described may influence whether prospective clients understand treatment mainly as a set of techniques or as a process that also involves empathy, acceptance, and collaboration. These results point to the value of carefully designed first-contact materials, intake documents, and consent procedures, especially in culturally diverse settings.

## Data Availability

The raw data supporting the conclusions of this article will be made available by the authors, without undue reservation.
