# Supplementary material for: Pre-treatment consent framing shapes how laypeople evaluate CBT: a cross-cultural comparison of China and the United States
Source: Front Psychol. 2026 Sep 3;17:1909096. doi: 10.3389/fpsyg.2026.1909096 (PMC13582562; doi:10.3389/fpsyg.2026.1909096)
Supplement: Supplementary file 1 [file Supplementary_file_1.DOCX]

**Supplementary Materials**

Pre-Treatment Consent Framing Shapes How Laypeople Evaluate CBT: A Cross-Cultural Comparison of China and the United States

[Author Names]

**Contents**

Figure S1. Supplementary Interaction and Simple Effects Plots for Non-Significant Variables

Figure S2. Informed Consent Subgroups Attitude Network

Figure S3. Centrality Comparison Across Informed Consent Subgroups

Table S1. Simple Effects of Information Type Within Each Cultural Group

Table S2. IS-RSA Results for Weighted Combination of Culture and Condition Predictors

Table S3 Demographic Characteristics of the Chinese and U.S. Samples and Between-Group Comparisons

Appendix A: Disclosure Scenarios

Appendix B: Descriptive Statements about Common and Specific Factors

**Figure S1**. Supplementary Interaction and Simple Effects Plots for Non-Significant Variables. Columns alternate between interaction line plots (a, c, e, g, left) and simple effects bar charts (b, d, f, h, right) for Empathy, Positive Regard, Expectancy, and CBT Techniques.

**Figure S2**. Informed Consent Subgroups Attitude Network. (a) Eastern attitude network of Informed Consent Subgroups. (b) Western attitude network of Informed Consent Subgroups.

**Figure S3**. Centrality Comparison Across Informed Consent Subgroups. (a) Eastern sample strength centrality by condition. (b) Western sample strength centrality by condition.

**Table S1. Simple Effects of Information Type Within Each Cultural Group**

| DV | Simple_Effect | Eastern  (M±SD) | Western  (M±SD) | t | | p |
| --- | --- | --- | --- | --- | --- | --- |
| Empathy | Culture at Standard | 3.39 ±1.38 | 3.18 ±1.29 | | 1.14 | 0.252696 |
| Empathy | Culture at Enhanced | 3.75 ±1.23 | 3.73 ±1.28 | | 0.11 | 0.912045 |
| Empathy | Culture at None | 3.84 ±1.24 | 3.33 ±1.30 | | 2.77 | 0.005765 |
| Working Alliance | Culture at Standard | 2.83 ±1.35 | 3.56 ±1.02 | | -3.93 | 0.000097 |
| Working Alliance | Culture at Enhanced | 3.36 ±1.32 | 3.40 ±1.32 | | -0.22 | 0.828808 |
| Working Alliance | Culture at None | 3.21 ±1.38 | 3.29 ±1.46 | | -0.43 | 0.668107 |
| Positive Regard | Culture at Standard | 2.62 ±1.17 | 2.22 ±1.08 | | 2.29 | 0.022634 |
| Positive Regard | Culture at Enhanced | 3.04 ±1.37 | 2.92 ±1.31 | | 0.69 | 0.492645 |
| Positive Regard | Culture at None | 2.90 ±1.23 | 2.59 ±1.29 | | 1.81 | 0.070266 |
| Expectancy | Culture at Standard | 2.36 ±1.26 | 2.00 ±1.23 | | 1.98 | 0.048442 |
| Expectancy | Culture at Enhanced | 2.43 ±1.29 | 2.30 ±1.31 | | 0.72 | 0.470066 |
| Expectancy | Culture at None | 2.19 ±1.28 | 2.37 ±1.31 | | -1 | 0.318603 |
| CBT Techniques | Culture at Standard | 3.80 ±1.43 | 4.04 ±1.29 | | -1.21 | 0.227857 |
| CBT Techniques | Culture at Enhanced | 2.41 ±1.39 | 2.65 ±1.41 | | -1.17 | 0.241088 |
| CBT Techniques | Culture at None | 2.85 ±1.44 | 3.41 ±1.40 | | -2.86 | 0.004451 |

DV = Dependent Variable, M = Mean, SD = Standard Deviation.

**Table S2. IS-RSA Results for Weighted Combination of Culture and Condition Predictors**

| **α (Culture)** | **α (Info)** | **ρ** | **Fisher-z** | **p (raw)** | **p (Bonferroni)** |
| --- | --- | --- | --- | --- | --- |
| 0.00 | 1.00 | 0.0333 | 0.0333 | 0.000200 | 0.004199 |
| 0.05 | 0.95 | 0.0361 | 0.0361 | 0.000200 | 0.004199 |
| 0.10 | 0.90 | 0.0361 | 0.0361 | 0.000200 | 0.004199 |
| 0.15 | 0.85 | 0.0361 | 0.0361 | 0.000200 | 0.004199 |
| 0.20 | 0.80 | 0.0361 | 0.0361 | 0.000200 | 0.004199 |
| 0.25 | 0.75 | 0.0361 | 0.0361 | 0.000200 | 0.004199 |
| 0.30 | 0.70 | 0.0361 | 0.0361 | 0.000200 | 0.004199 |
| 0.35 | 0.65 | 0.0361 | 0.0361 | 0.000200 | 0.004199 |
| 0.40 | 0.60 | 0.0361 | 0.0361 | 0.000200 | 0.004199 |
| 0.45 | 0.55 | 0.0361 | 0.0361 | 0.000200 | 0.004199 |
| 0.50 | 0.50 | 0.0348 | 0.0348 | 0.000200 | 0.004199 |
| 0.55 | 0.45 | 0.0310 | 0.0310 | 0.000200 | 0.004199 |
| 0.60 | 0.40 | 0.0310 | 0.0310 | 0.000200 | 0.004199 |
| 0.65 | 0.35 | 0.0310 | 0.0310 | 0.000200 | 0.004199 |
| 0.70 | 0.30 | 0.0295 | 0.0295 | 0.000200 | 0.004199 |
| 0.75 | 0.25 | 0.0295 | 0.0295 | 0.000200 | 0.004199 |
| 0.80 | 0.20 | 0.0295 | 0.0295 | 0.000200 | 0.004199 |
| 0.85 | 0.15 | 0.0295 | 0.0295 | 0.000200 | 0.004199 |
| 0.90 | 0.10 | 0.0295 | 0.0295 | 0.000200 | 0.004199 |
| 0.95 | 0.05 | 0.0295 | 0.0295 | 0.000200 | 0.004199 |
| 1.00 | 0.00 | 0.0156 | 0.0156 | 0.000200 | 0.004199 |

Info = Information type.

**Table S3 Demographic Characteristics of the Chinese and U.S. Samples and Between-Group Comparisons**

| Variable | China  （n=297） | U.S  (n=303 ) | t/$\chi^{2}$ | | *p* |
| --- | --- | --- | --- | --- | --- |
| Gender(male/ female/no report) | 102/195/0 | 132/170/1 | 6.49 | 0.036 | |
| Age(M±SD) | 28.14±9.97 | 31.82±11.08 | -4.28 | ＜0.001 | |
| Education level |  |  | 29.22 | ＜0.001 | |
| *High school or below* | 35 | 22 |  |  | |
| *Associate degree* */ College diploma* | 21 | 61 |  |  | |
| *Bachelor's degree* | 145 | 156 |  |  | |
| *Postgraduate degree* | 96 | 64 |  |  | |

To assess the comparability of the two national samples, we compared their gender, age, and educational characteristics (Table S1). Their demographic profiles were broadly similar, although some modest differences were found. The Chinese sample included a slightly higher proportion of women than the U.S. sample (65.7% vs. 56.1%), χ²(1, N = 600) = 6.49, *p* = .036, Cramér’s V = 0.104. Chinese participants were about 3.7 years younger on average (M = 31.82 vs. 28.14), t(598) = −4.28, p < .001, Cohen’s d = 0.35. Educational distributions also differed, χ²(3, N = 600) = 29.22, p < .001, Cramér’s V = .221. However, most participants in both samples held at least a bachelor’s degree. The variation was mainly found in the some-college/associate and graduate-level categories, rather than reflecting a clear overall gap in educational attainment.

**Appendix A: Disclosure Scenarios**

**Standard CBT Disclosure Scenario**

Someone feeling very low visits the doctor. After making a diagnosis of depression, the doctor recommends a form of psychotherapy called cognitive behavioral therapy (CBT). The client is unsure what CBT is but the doctor explains how it works and provides the following information in a leaflet. The doctor asks the client to read it:

“Cognitive behavioral therapy (‘CBT’) is a form of psychotherapy or talking treatment which is commonly used to treat depression. It helps you deal with your problems more effectively. CBT focuses on a person’s thoughts and beliefs and how they influence a person’s mood and actions. Negative thoughts and feelings can trap you in a vicious cycle. CBT aims to help you break the vicious cycle of unhealthy thinking patterns. The therapist helps you to identify distorted thinking patterns that may be making you depressed. Your therapist will help you to recognize inaccurate thoughts and change your thinking and behavior accordingly.”

The client decides to undergo CBT therapy.

**Common Factors Disclosure Scenario**

Someone feeling very low visits the doctor. After making a diagnosis of depression, the doctor recommends a form of psychotherapy called cognitive behavioral therapy (CBT). The client is unsure what CBT is but the doctor explains how it works and provides the following information in a leaflet. The doctor asks the client to read it:

“Cognitive behavioral therapy (‘CBT’) is a form of psychotherapy or talking treatment which is commonly used to treat depression. The therapist helps you to identify distorted thinking patterns that may be making you depressed. However, CBT works best for those clients who can agree on the tasks and goals of therapy and form a good working relationship with their therapist. It is also more effective when the therapist is empathetic (understands the client’s perspective and feelings) and supports and encourages the client throughout their time in therapy.”

The client decides to undergo CBT therapy.

**No Disclosure Scenario**

Someone feeling very low visits the doctor. After making a diagnosis of depression, the doctor recommends a form of psychotherapy called cognitive behavioral therapy (CBT). The client is unsure what CBT is but decides to undergo CBT anyway.

**Appendix B: Descriptive Statements about Common and Specific Factors**

**Instructions:**

Below are several descriptive statements regarding different factors involved in Cognitive Behavioral Therapy (CBT). Please rate how important you believe each factor is for a successful treatment.

**Please indicate your rating using the following 5-point scale:**

1 = Not at all important, 2 = Slightly important, 3 = Moderately important, 4 = Very important, 5 = Extremely important.

**Common Factors – Empathy**

The therapist understands the client’s problems and feelings and can see things from the client’s perspective. The therapist communicates this understanding and shows concern for the client.

**Common Factors – Working Alliance**

The therapist and the client agree about the goals and tasks of therapy. The therapist and client collaborate and form a good working alliance in trying to achieve these goals.

**Common Factors – Positive Regard**

The therapist has a warm demeanor and is reassuring toward the client. The therapist accepts that the client is finding things difficult, and encourages and supports the client.

**Common Factors – Positive Expectations**

The client expects CBT to be an effective treatment for depression. The therapist also shares the belief that CBT is an effective form of treatment for clients who are depressed.

**Specific Factors of CBT**

The therapist applies the specific principles of CBT with the client. The therapist teaches the client how to identify, evaluate and respond to depressive thoughts and behaviors using the theory and methods of CBT.
